# First Images of a Three-Layer Compton Telescope Prototype for Treatment Monitoring in Hadron Therapy

**DOI:** 10.3389/fonc.2016.00014

**Published:** 2016-02-02

**Authors:** Gabriela Llosá, Marco Trovato, John Barrio, Ane Etxebeste, Enrique Muñoz, Carlos Lacasta, Josep F. Oliver, Magdalena Rafecas, Carles Solaz, Paola Solevi

**Affiliations:** ^1^Instituto de Física Corpuscular (IFIC-CSIC/UVEG), Valencia, Spain

**Keywords:** Compton camera, Compton telescope, hadron therapy, treatment monitoring, LaBr_3_

## Abstract

A Compton telescope for dose monitoring in hadron therapy is under development at IFIC. The system consists of three layers of LaBr_3_ crystals coupled to silicon photomultiplier arrays. ^22^Na sources have been successfully imaged reconstructing the data with an ML-EM code. Calibration and temperature stabilization are necessary for the prototype operation at low coincidence rates. A spatial resolution of 7.8 mm FWHM has been obtained in the first imaging tests.

## 1 Introduction

Hadron therapy allows a more precise delivery of charged particles in the tumor region as compared to photons. In order to fully exploit the benefits of this technique and reduce the safety margins applied, the dose administration requires accurate verification of the treatment delivery in real time. PET techniques currently employed suffer from some limitations such as low efficiency or the fact that the metabolic processes carry away the activity (biological washout). Also, positron production does not follow irradiation immediately and the difficulties of integrating the monitoring device with the treatment delivery make it hard to combine simultaneous treatment and monitoring. Different ways of achieving real-time monitoring are under investigation, employing other types of secondary particles emitted by the tissue after irradiation, such as prompt gamma-rays, which are emitted by the excited tissue nuclei within nanoseconds after irradiation (1). The ENVISION^1^ European project has addressed this problem by improving PET systems and developing novel devices for the detection of prompt gammas.

Collimated systems (2, 3) and Compton cameras (4–7) are possible alternatives to image such gamma-rays, with energies mainly in the range of 0.5 to about 10 MeV. Prompt gamma timing techniques are also being investigated (8). Such systems have proven their ability to distinguish range shifts in beam tests. Compton cameras can offer higher efficiency than collimated cameras, as well as 3D imaging. For the construction of Compton cameras, different detector materials and geometries are being investigated, including silicon detectors, CZT, gas chambers, or scintillator detectors. Two approaches are followed: two-layer Compton cameras, with the traditional approach of a scatter detector followed by an absorber detector, and multiple-layer Compton cameras, requiring at least three interactions in three detectors. Two-layer Compton cameras have higher efficiency, but they rely on the knowledge of the incoming gamma-ray energy or on full absorption on the second detector for the determination of its energy. In this application, full absorption is difficult due to the high energies of the gamma-rays and the energy spectrum is broad and continuous up to high energies. The detection of three interactions on three detector layers fully determines the energy of the incoming gamma-ray, improving detector resolution but decreasing efficiency by an order of magnitude with respect to the two-layer option.

A Compton telescope (multilayer Compton camera) based on several planes of continuous LaBr_3_ crystals coupled to silicon photomultiplier (SiPM) arrays is under development at IFIC, Valencia (9). We aim to combine both two- and three-layer modalities in one system to maximize resolution and efficiency. In addition, we have developed a method to estimate the energy from two-layer events. The choice of LaBr_3_ as scintillator detector makes it possible to achieve excellent energy and timing resolution. LaBr_3_ has been employed in Compton telescopes for gamma-ray astronomy in the megaelectronvolt range (10). SiPMs are fast and their reduced thickness minimizes the probability of gamma-rays interacting in the photodetector. This facilitates gamma-rays to escape one detector and reach the next one. The whole system is light, portable, scalable, and easy to operate. We have assembled a three-layer version of the system. In this article, we present the first images obtained with the three-layer prototype, assessing the imaging capabilities of the device.

## 2 Materials and Methods

### 2.1 Prototype Description

**Figure 1:**
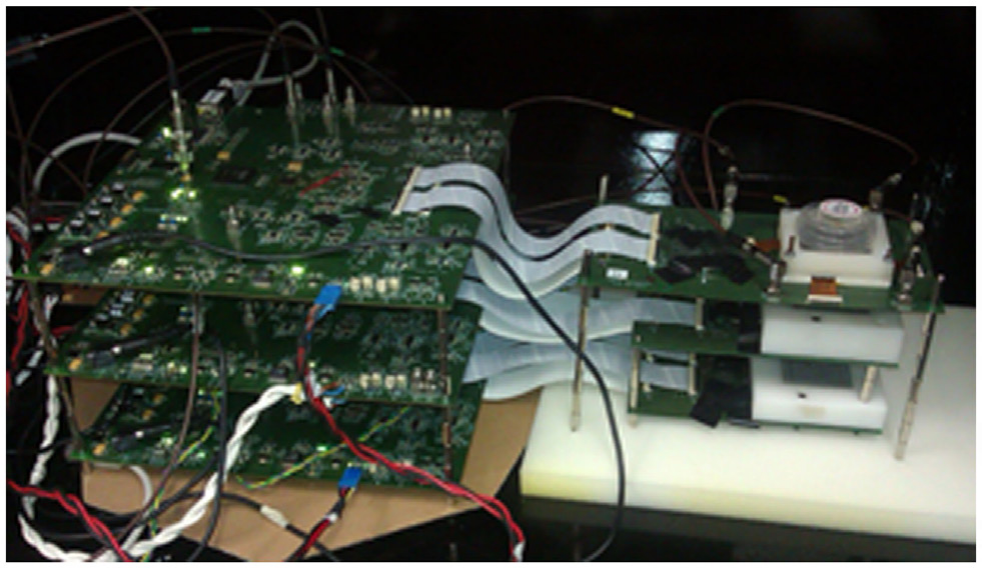
**Three-layer Compton telescope prototype and readout electronics**.

The prototype consists of three detector layers, each one attached to a readout electronics board (Figure 1) (11). The first layer is made of a 27.2 mm × 26.8 mm × 5 mm LaBr_3_ crystal coupled to four Hamamatsu MPPC S11830-3340MF monolithic arrays, with 4 × 4 pixels each. The arrays are biased individually. The second and third layers are composed of crystals of size 32 mm × 36 mm and thickness of 5 and 10 mm, respectively, coupled to four S11064-050P(X1)arrays with a common bias for all of them.

The readout of each plane is done with a custom-made data acquisition (DAQ) system that drives the 64-channel ASIC VATA64HDR16 (12). The DAQ board is equipped with an FPGA that controls the acquisition process, an 8-bit ADC (analog-to-digital converter) to digitize the data, and it is connected to a PC through Ethernet connection. The ASIC allows individual adjustment of the bias voltage of the 64 SiPM elements in the array through input DACs (digital-to-analog converters) in each channel.

### 2.2 Detector Characterization

The three detector layers have been characterized independently by taking data with radioactive sources of different energies (^22^Na, ^137^Cs, and ^60^Co). The light generated in the crystal by the gamma-rays is detected by the 64-pixel elements of the SiPM array. For each event, the signals produced in each of the pixels are digitized and stored for data analysis.

The uniformity of the detector response has also been evaluated. The ^22^Na source is placed 15 cm away from the detector in order to ensure a uniform illumination. The signals in each channel are histogrammed for all the events acquired, and the average signal per channel is assumed to be constant for a high number of events (>10,000) (13). This way, the differences in response among channels can be appreciated. In order to equalize the response, the bias voltage per channel is adjusted through the ASIC input DACs.

In order to obtain the energy spectra, for each event the 64 ADC values of the SiPM signals are summed up and histogrammed. A calibration curve is obtained by taking data with sources of different energies, fitting a Gaussian function to the photopeaks in the spectra in order to determine the peak position in ADC counts, and plotting the peak position versus the source energy.

The determination of the interaction position in the crystal is carried out with the method described in Ref. (14), which is based on a model of the light distribution in the photodetector, taking into account both the photons that reach it directly and those that are first reflected on the crystal sides. In order to determine the intrinsic spatial resolution, data are taken with a ^22^Na source placed at different positions of the detector surface. The source is electronically collimated by operating the detector in time coincidence with a small detector, restricting the position at which the photons interact.

The current–voltage characteristics of the SiPM depend on the operating temperature. This is mainly due to the change in the breakdown voltage of the SiPM, which results in a different overvoltage for a given bias voltage applied to the detector. The variations of the photopeak position of the energy spectra with temperature have been studied. ^22^Na energy spectra are taken with the detectors in a climatic chamber, at different temperatures. A figure representative of the detector gain is calculated from the two photopeak positions of ^22^Na in each case.

### 2.3 Prototype Operation

The three detector layers have been assembled in order to work in time coincidence. The trigger signal generated by each detector is sent to a NIM coincidences unit. The coincidence is given by the overlap of the trigger signals of the three detectors, which is 25 ns wide. The threshold applied to the detectors is around 50 keV. The output coincidence signal is sent back to each of the DAQ boards in order to start the data acquisition.

The distance from the source to the first layer is 35 mm. The distances from the first to the second layer and from the second to the third are 60 and 65 mm, respectively. Coincidence data with the three layers have been taken placing the system inside a climatic chamber in order to maintain the temperature constant (the measurement was done at 25.5°C) and avoid temperature variations during data acquisition.

Data are taken with a ^22^Na source of 0.25 mm active diameter and 700 kBq activity. The data recorded in the three detectors are calibrated and summed up for each event. The energies and interaction positions in the three layers are the input of the image reconstruction code.

### 2.4 Image Reconstruction

For image reconstruction in Compton cameras, conventional two-interaction events require to know the energy of the incoming photon or full absorption in the second detector in order to obtain the cone surface defined by all the possible photon trajectories. In hadron therapy monitoring, this is not possible due to the wide emission spectrum and the high photon energies. To overcome this limitation, the incoming photon energy can be estimated during the image reconstruction process, *spectral reconstruction* (15). However, havin g three layers allows us to access to three-interaction events, which convey enough information to directly determine this energy and, therefore, the associated cone surface.

An image reconstruction software based on the statistical iterative algorithm maximum likelihood-expectation maximization (ML-EM) has been developed. These data are acquired in coincidence list-mode and the interaction positions and energies are directly used (not histogrammed) for avoiding resolution loss. The reconstruction code (16) implements the above-mentioned strategy to reconstruct the three-interaction events data.

## 3 Results

### 3.1 Detector Characterization

The detectors employed in the prototype have been characterized in terms of energy and spatial resolution.

**Figure 2:**
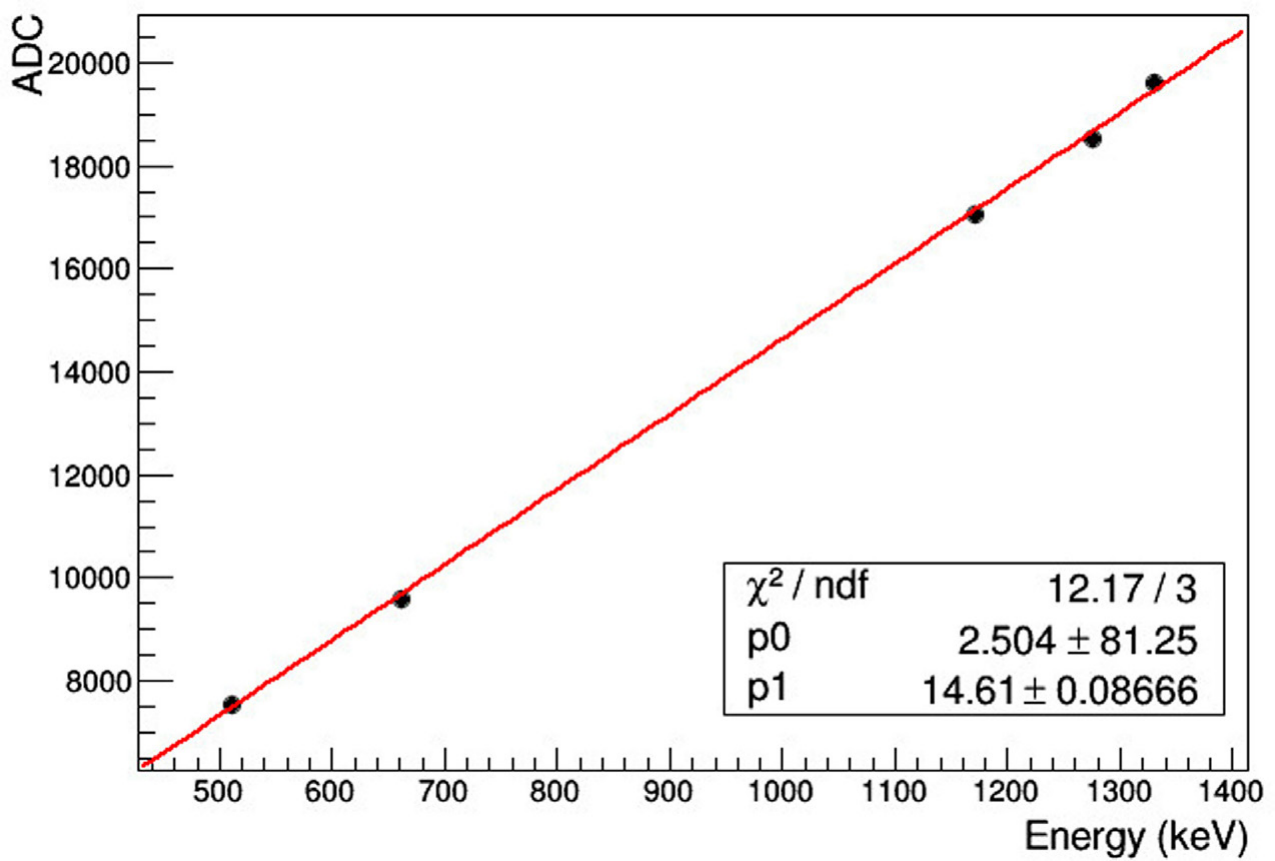
**Photopeak position vs. source energy for detector calibration**.

**Figure 3:**
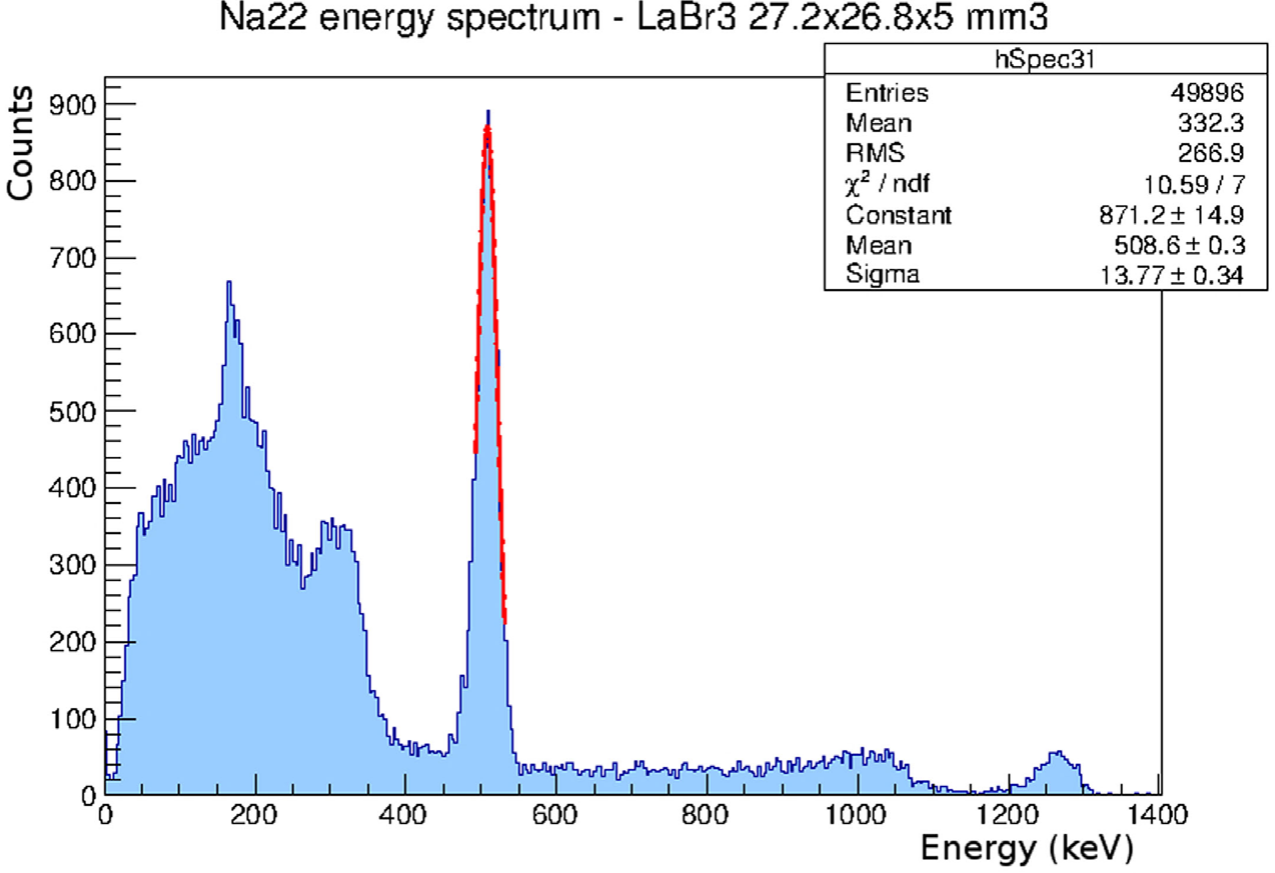
**^22^Na energy spectrum obtained with the first detector**. The energy resolution is 6.4% FWHM at 511 keV.

The results of the first detector calibration are shown in Figure 2, where it can be seen that the response is linear up to 1.33 MeV. Similar results are obtained with the other two detectors. Figure 3 shows a ^22^Na energy spectrum obtained also with the first detector. A Gaussian fit to the 511 keV photopeak results in an energy resolution of 6.4% FWHM. An energy resolution of 7.4% FWHM and 7.2% FWHM at 511 keV has been obtained with the second and third layers, respectively.

The intrinsic spatial resolution achieved with the three detectors is of the order of 1 mm FWHM (17). The uniformity of the pixel response achieved applying the DAC corrections is around 5% in the first detector, and around 10% in the second and third detectors. The difference is due to the fact that the first detector employs monolithic arrays that have a more uniform response within each array, and the four of them can be biased individually, adjusting better the four bias reference voltages.

**Figure 4:**
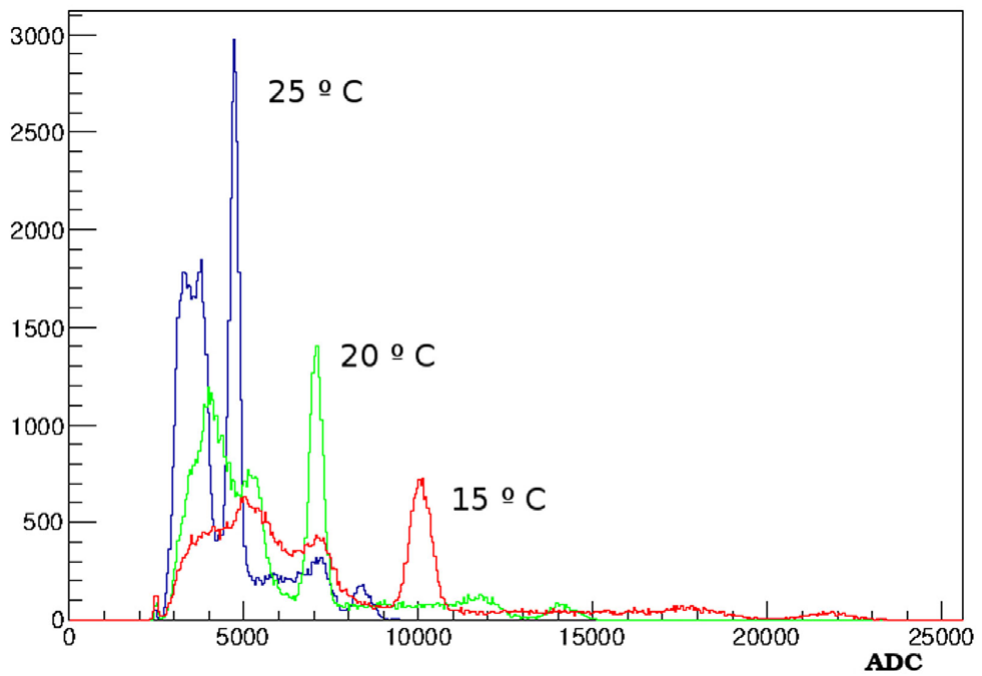
**^22^Na spectra obtained at different temperatures where one can see the gain variation**.

The effects of temperature variations are shown in Figure 4, which shows ^22^Na energy spectra taken at different temperatures. The difference in gain can be clearly appreciated.

**Figure 5:**
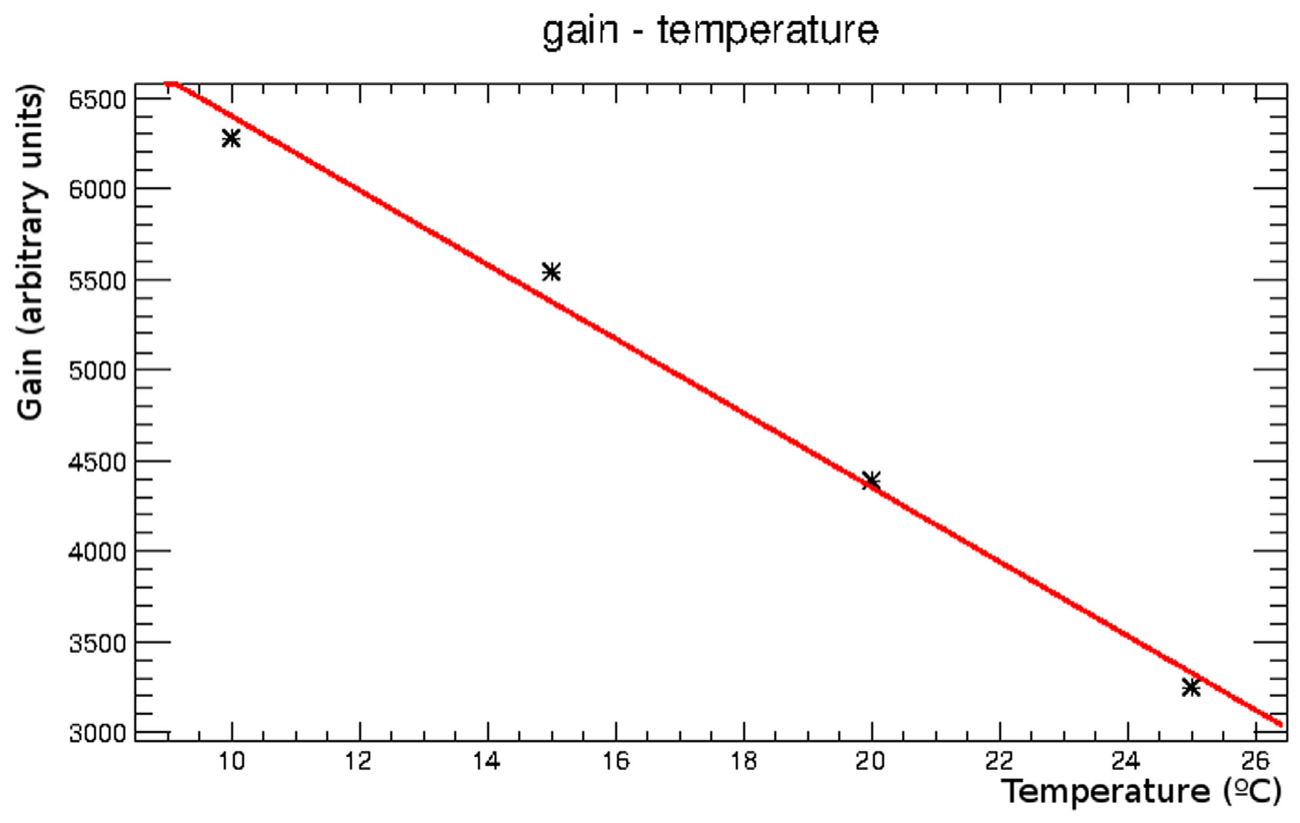
**Gain obtained from the calibration curves at different temperatures, plotted as a function of temperature**.

In Figure 5, the gain values obtained from the energy spectra are plotted versus temperature. The gain decrease with temperature is about 5%/°C.

### 3.2 Prototype Results

**Figure 6:**
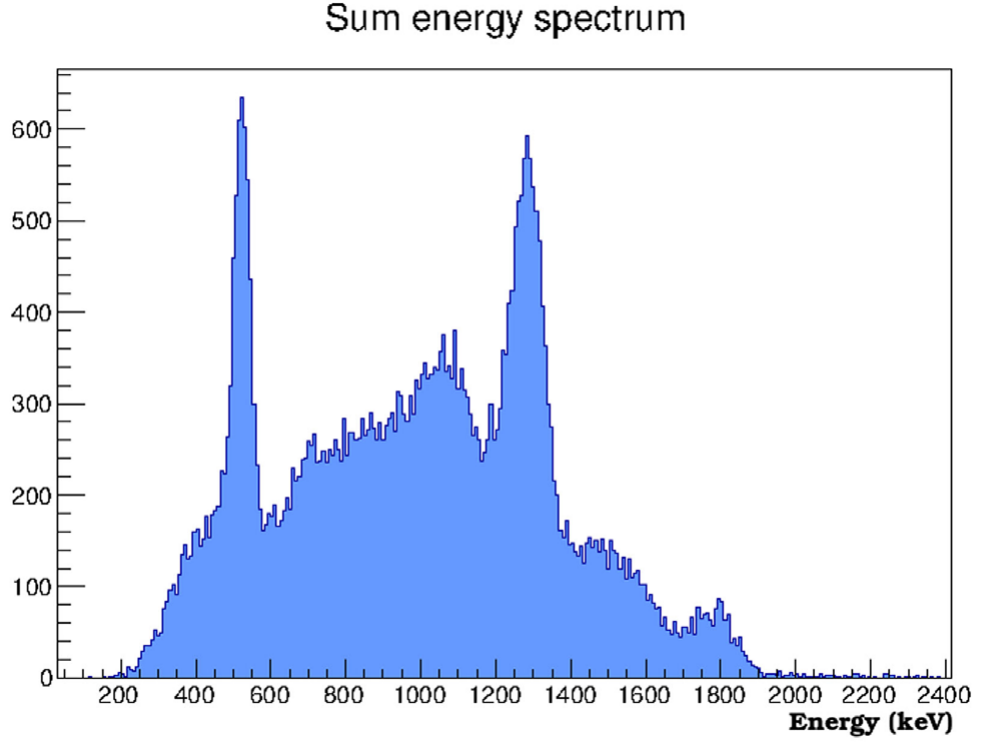
**Calibrated energy spectrum obtained by summing the energies recorded in the three detectors in coincidence for each event**.

The ^22^Na energy spectrum corresponding to the sum of the energies recorded in the three detector layers in coincidence in each event is shown in Figure 6. The two ^22^Na photopeaks (511 and 1275 keV) can be observed. A sum peak of the previous two due to accidental coincidences can also be seen. The count rate with the tested geometry is 0.3 events/s and the calculated efficiency is about 7 × 10^−6^.

**Figure 7:**
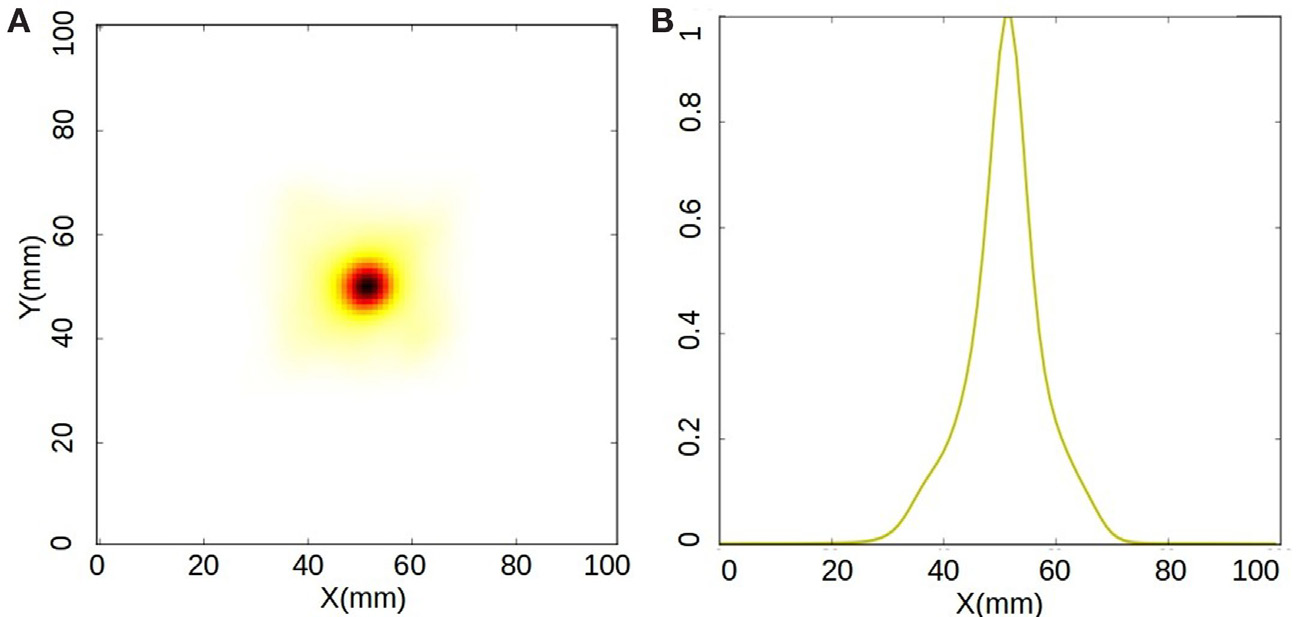
**(A)** 2D view of the reconstructed image of the ^22^Na source at the source plane. **(B)** Profile through the peak of the plot. The resolution is 7.8 mm FWHM.

The processed data are employed for image reconstruction. Figure 7A shows a 2D view of a reconstructed image with a total energy cut between 800 and 1400 keV in the sum spectrum, after 30 iterations. Figure 7B shows a profile along the x axis through the maximum of the reconstructed image. A Gaussian fit to the profile results in a spatial resolution of 7.8 mm FWHM for the geometry employed and the cuts applied. In the tests reported here, it was not possible to obtain an image employing the data corresponding to the 511 keV photopeak.

## 4 Discussion and Future Work

A Compton telescope composed of three layers of LaBr_3_ crystals coupled to SiPM arrays has been successfully constructed and operated. The energy resolution obtained with the newest detector (first detector) 6.4% FWHM at 511 keV is closer to the one specified by the crystal manufacturer and measured by us with a PMT, 3.5% FWHM (17) and compatible with other measurements with SiPMs (18). Further improvements of the SiPM array pixel uniformity and photon detection efficiency in the LaBr_3_ peak emission wavelength (380 nm), together with an improved detector coupling should make it possible to achieve the excellent energy resolution expected with this kind of scintillator crystal. The intrinsic spatial resolution of the detectors, close to 1 mm FWHM, is appropriate for the application. The timing resolution must also be characterized and brought close to 1 ns FWHM in order to reject the neutron background (19). The response of the detectors to temperature variations has been studied, and the temperature calibration can be applied to compensate for temperature changes when temperature control is not possible.

Even with this non-optimized setup, it has been possible to obtain an image of a ^22^Na source in the laboratory. An image reconstruction code has been developed, and it is ready for its use. The resolution of the reconstructed image in this first attempt is 7.8 mm FWHM at 35 mm distance from the first detector.

The spatial resolution achieved should still be improved in order to determine the position of the distal falloff with few millimeter accuracy, as it is required for hadron therapy monitoring (20).

As expected, the experimental results obtained in this first test are behind similar systems in a more advanced development status. Comparison of the results with other approaches is not possible at this point due to the different sizes, configurations, and geometries. Work is being carried out to optimize the system results and estimate its potential capabilities.

A Monte Carlo simulation code has also been developed to optimize the detector configuration and determine the necessary improvements for its application to hadron therapy monitoring.

## Author Contributions

GL has supervised the work, helped to carry it out, and wrote the article. MT has set up the device and done the measurements described. All other authors have contributed to some parts of the work: CS, electronics; EM and AE, analysis software; CL, acquisition software; JB, measurements; PS and JO, image reconstruction; MR, supervision and guidance for the image reconstruction part of the work.

## Conflict of Interest Statement

The authors declare that the research was conducted in the absence of any commercial or financial relationships that could be construed as a potential conflict of interest.

## Footnotes

^1^http://envision.web.cern.ch/ENVISION/

## References

[1] Min C Kim C Youn M Kim J Prompt gamma measurements for locating dose falloff region in proton therapy. Appl Phys Lett (2006) 89:183511–3. 10.1063/1.2378561

[2] Kim D Yim H Kim J-W Pinhole camera measurements of prompt gamma-rays for detection of beam range variation in proton therapy. J Korean Phys Soc (2009) 55:1673–6. 10.3938/jkps.55.1673

[3] Roellinghoff F Benilov A Dauvergne D Dedes G Freud N Janssens G Real-time proton beam range monitoring by means of prompt-gamma detection with a collimated camera. Phys Med Biol (2014) 59:1327–38. 10.1088/0031-9155/59/5/1327 24556873

[4] Richard M-H Chevallier M Dauvergne D Freud N Henriquet P Foulher FL Design guidelines for a double scattering Compton camera for prompt-gamma imaging during ion beam therapy: a Monte Carlo simulation study. IEEE Trans Nucl Sci (2011) 58:87–94. 10.1109/TNS.2010.2076303

[5] Kormoll T Fiedler F Schone S Wustemann J Zuber K Enghardt W A Compton imager for in-vivo dosimetry of proton beams – a design study. Nucl Instrum Methods Phys Res A (2011) 626:114–9. 10.1016/j.nima.2012.01.072

[6] Kurosawa S Kubo H Ueno K Kabuki S Iwaki S Takahashi M Prompt gamma detection for range verification in proton therapy. Curr Appl Phys (2012) 12:364–8. 10.1016/j.cap.2011.07.027

[7] Polf JC Avery S Mackin DS Beddar S. Imaging of prompt gamma rays emitted during delivery of clinical proton beams with a Compton camera: feasibility studies for range verification. Phys Med Biol (2015) 60:7085–99. 10.1088/0031-9155/60/18/7085 26317610

[8] Golnik C Hueso-González F Mueller A Dendooven P Enghardt W Fiedler F Range assessment in particle therapy based on prompt-ray timing measurements. Phys Med Biol (2014) 59:5399–422. 10.1088/0031-9155/59/18/5399 25157685

[9] Llosá G Cabello J Callier S Gillam JE Lacasta C Rafecas M First Compton telescope prototype based on continuous LaBr3-SiPM detectors. Nucl Instrum Methods Phys Res A (2013) 718:130–3. 10.1016/j.nima.2012.08.074

[10] Bloser PF Ryan JM Legere JS Julien M Bancroft CM McConnell ML A new low-background Compton telescope using LaBr3 scintillator. In: Proc. SPIE 7435, UV, X-Ray, and Gamma-Ray Space Instrumentation for Astronomy XVI (2009). 10.1117/12.826191

[11] Trovato M Solevi P Torres-Espallardo I Gillam J Lacasta C Rafecas M A three layer Compton telescope for dose monitoring in hadron therapy. IEEE NSS MIC Seattle, WA: IEEE (2014).

[12] Stankova V Barrio J Gillam JE Lacasta C Rafecas M Solaz C Multichannel DAQ system for SiPM matrices. Nuclear Science Symposium and Medical Imaging Conference (NSS/MIC). Anaheim, CA: IEEE (2012). p. 1069–71. 10.1109/NSSMIC.2012.6551270

[13] Barrio J Etxebeste A Lacasta C Muñoz E Oliver J Solaz C Performance of vata64hdr16 ASIC for medical physics applications based on continuous crystals and SiPMS. J Instrum (2015) 10:12001 10.1088/1748-0221/10/12/P12001

[14] Li Z Wedrowski M Bruyndonckx P Vandersteen G Nonlinear least-squares modelling of 3d interaction position in a monolithic scintillator block. Phys Med Biol (2010) 55:6515–32. 10.1088/0031-9155/55/21/012 20959686

[15] Xu D He Z Gamma-ray energy-imaging integrated spectral deconvolution. Nucl Instrum Methods Phys Res A (2007) 574:98–109. 10.1016/j.nima.2007.01.171

[16] Gillam JE Oliver JF Torres-Espallardo I Lacasta C Llosá G Trovato M Simulated one pass listmode for fully 3d image reconstruction of a Compton camera data. IEEE NSS MIC Conference Record (2012). p. 3298–305. 10.1109/NSSMIC.2012.6551752

[17] Llosá G Cabello J Gillam JE Lacasta C Oliver JF Rafecas M Second LaBr3 Compton telescope prototype. ANIMMA 2013 Conference Record Marseille: IEEE (2013). p. 1–4.

[18] Seifert S van Dam HT Huizenga J Vinke R Dendooven P Lohner H Monolithic LaBr3:Ce crystals on silicon photomultiplier arrays for time-of-flight positron emission tomography. Phys Med Biol (2012) 57:2219–33. 10.1088/0031-9155/57/8/2219 22455977

[19] Biegun AK Seravalli E Lopes PC Rinaldi I Pinto M Oxley DC Time-of-flight neutron rejection to improve prompt gamma imaging for proton range verification: a simulation study. Phys Med Biol (2012) 57:6429–44. 10.1088/0031-9155/57/20/6429 22996154

[20] Moteabbed M España S Paganetti H. Monte Carlo patient study on the comparison of prompt gamma and pet imaging for range verification in proton therapy. Phys Med Biol (2011) 56:1063–82. 10.1088/0031-9155/56/4/012 21263174

